# Heat stress response in Chinese cabbage (*Brassica rapa* L.) revealed by transcriptome and physiological analysis

**DOI:** 10.7717/peerj.13427

**Published:** 2022-05-25

**Authors:** Lei Zhang, Yun Dai, Lixin Yue, Guohu Chen, Lingyun Yuan, Shifan Zhang, Fei Li, Hui Zhang, Guoliang Li, Shidong Zhu, Jinfeng Hou, Xiaoyan Tang, Shujiang Zhang, Chenggang Wang

**Affiliations:** 1 College of Horticulture, Vegetable Genetics and Breeding Laboratory, Anhui Agricultural University, Hefei, China; 2 Institute of Vegetables and Flowers, Chinese Academy of Agricultural Sciences, Beijing, China; 3 Institute of Vegetables, Shandong Academy of Agricultural Sciences, Jinan, China

**Keywords:** Chinese cabbage, Heat stress, Transcriptome, ROS, Physiology

## Abstract

High temperatures have a serious impact on the quality and yield of cold-loving Chinese cabbage, which has evolved to have a unique set of stress mechanisms. To explore the relationship between these mechanisms and the heat-tolerance of Chinese cabbage, the physiological indicators of the heat-tolerant ‘268’ line and heat-sensitive ‘334’ line were measured. Under heat stress, the proline (Pro), soluble sugar (SS), and superoxide dismutase (SOD) indexes of the ‘268’ line increased significantly. When additionally using transcriptome analysis, we found that the identified 3,360 DEGs were abundantly enriched in many metabolic pathways including ‘plant hormone signal transduction’, ‘carbon metabolism’, and ‘glycolysis/gluconeogenesis’. Dynamic gene expression patterns showed that *HKL1* in Cluster 15 may be a key factor in the regulation of sugar homeostasis. The interaction network screened four ABA-related genes in Cluster 15, suggesting that high temperatures lead to changes in hormonal signaling, especially an increase in ABA signaling. Compared with the ‘334’ line, the expressions of *Prx50*, *Prx52*, *Prx54*, *SOD1*, and *SOD2* in the ‘268’ line were significantly upregulated, and these genes were actively involved in the reactive oxygen species (ROS) scavenging process. In summary, our results revealed the relationship between plant heat tolerance, physiology, and biochemistry and may also provide ideas for the future development of high-quality and heat-tolerant Chinese cabbage germplasm resources.

## Introduction

High temperatures caused by global climate change have a serious impact on plant growth and development. Heat stress makes plant leaves curl and yellow, scorches edges, and wilts leaves, which greatly reduce crop yield (Sharma et al., 2016). Moreover, high temperatures lead to the production of reactive oxygen species (ROS) and a series of stress responses in plants. An excessive accumulation of ROS weakens the antioxidant capacity of plants, reduces photosynthesis, and destroys the activity of biological macromolecules (El-Esawi & Alayafi, 2019). High temperature environments enhance transpiration and evaporation processes, resulting in plant dehydration and even death (Locato, de Pinto & De Gara, 2009). Therefore, plants have evolved complex stress mechanisms in response to high temperatures (Qi et al., 2021).

Plants use a complex set of physiological and molecular regulation mechanisms to resist high temperatures (Guan et al., 2013; Song et al., 2014). These pathway responses can effectively reduce the damage caused by heat stress with an increase in the activity of free radical scavenging enzymes such as peroxidase (POD), catalase (CAT) and superoxide dismutase (SOD), and the accumulation of malondialdehyde (MDA), free amino acids, soluble sugars (SS), and proline (Pro) (Bita & Gerats, 2013; Chen & Li, 2016). Additionally, the levels of some plant hormones, such as ethylene and abscisic acid (ABA), increase, while the levels of auxin (IAA), cytokinin (CTK), and gibberellin (GA) decrease under heat stress (Larkindale et al., 2005; Verma, Ravindran & Kumar, 2016). It has been reported that the involvement of ABA in the regulation of biochemical pathways after heat stress is necessary for plant survival (Wahid et al., 2007). Brassinosteroid (BR) can improve heat tolerance in rape, tomato, and other crops (Dhaubhadel et al., 1999). Under high temperatures, many signal transduction pathways are activated in plants. First, the heat stress response (HSR) involving Ca^2+^ and ROS is activated (Jacob, Hirt & Bendahmane, 2017). Heat shock transcription factors (HSFs) are the main regulatory factors involved in the HSR process (Liu et al., 2018). They initiate the expression of downstream gene heat shock proteins (HSPs) and activate the heat stress response (Jacob, Hirt & Bendahmane, 2017). In addition, HSPs can act as a molecular chaperone to protect protein folding and degradation and improve stress tolerance (Hartl & Hayer-Hartl, 2002).

Chinese cabbage (*Brassica rapa L*.) is a leafy vegetable from the genus *Brassica* in the family Cruciferae. Its cultivated area and consumption rank first among all vegetables grown in China (Dai et al., 2021; Qi et al., 2010). Chinese cabbage like to be cool with an appropriate temperature for the growing season of 15–20 °C, so it is usually planted in autumn. However, hot or abnormal weather leads to delayed growth, yellowing and wilting of leaves, and in severe cases, destroy the formation of leaf balls and an increase in susceptibility to infectious disease, which greatly reduces its quality and yield (Yue et al., 2021). Therefore, it is important to explore the mechanism of heat tolerance in cabbage through physiological and biochemical, as well as molecular, mechanism studies in order to develop new varieties of cabbage with high-temperature tolerance (Verma, Ravindran & Kumar, 2016).

The purpose of this study is to use physiological and biochemical indicators, RNA-seq, and a co-expression trend analysis for two different heat-tolerant lines of Chinese cabbage in order to more accurately determine the pathways and genes that may interact with heat stress and recovery conditions.

## Materials and Methods

### Plant material and treatment

Two Chinese cabbage highly inbred lines (heat-tolerant ‘268’ and the heat-sensitive ‘334’) were provided by the Chinese Academy of Agricultural Sciences, Beijing, China. Five hundred full and uniform seeds were selected for germination, sowing, and seedling raising. They were grown at 25 ± 2 °C with a photoperiod of 16 h light/8 h dark, a 150 µmol m^−2^ s^−1^ light intensity, and a humidity level of about 54% to avoid dehydration. After 30 days of growth, the ‘268’ and ‘334’ lines were separated into two groups: one was treated at 40 °C/30 °C (light/dark), and the other was treated at 25 °C (control). On the 10th day of treatment, the heat-stressed plants were transferred to the control environment for 4 d.

At 0 (CK), 4 (HT-4), 8 (HT-8), and 10 (HT-10) d of heat stress at 40 °C, and at 4 (RC-4) d after recovery treatment at 25 °C, three Chinese cabbage plants with the same level of growth (three biological replicates) were selected to sample the functional leaves, namely, the third leaf of each seedling center that was fully expanded from the inside to the outside. The samples were immediately pre-cooled in liquid nitrogen and then stored in an ultra-low temperature refrigerator at −80 °C for later RNA-seq. The sample used for measuring physiological indexes was selected from the functional leaves of the same part, cut into pieces, and mixed for later use.

### Physiological measurements

The differences in the physiological responses of the ‘268’ and ‘334’ lines under control and heat stress treatments were measured during CK, HT-4, HT-8, HT-10, and RC-4 periods. In accordance with the manufacturer’s instructions, the corresponding kits (Solarbio Life Sciences, Beijing, China) were selected to determine various physiological indicators. The Catalase (CAT) Activity Assay Kit (Cat#BC0200; Solarbio, Beijing, China), Superoxide Dismutase (SOD) Activity Assay Kit (Cat#BC0170; Solarbio, Beijing, China), Proline (Pro) Content Assay Kit (Cat#BC0290; Solarbio, Beijing, China), Malondialdehyde (MDA) Content Assay Kit (Cat#BC0020; Solarbio, Beijing, China), and Plant Soluble Sugar (SS) Content Assay Kit (Cat#BC0030; Solarbio, Beijing, China) were used for subsequent spectrophotometric measurements. Three biological replicates of each sample (*n* = 3) were assessed.

### Total RNA extraction, library preparation and assembly, and analysis

The heat-tolerant ‘268’ line and heat-sensitive ‘334’ line were used for testing. Total RNA was extracted using TRIzol reagent (Invitrogen, Carlsbad, CA, USA), and 30 cDNA libraries (two lines × five periods × three replicates) were constructed. The purity, concentration, and integrity of the RNA samples were tested using Nanodrop2000 (Shimadzu, Japan) and agarose gel electrophoresis. A total amount of 1 μg RNA per sample was used as input material for the RNA sample preparations. The Next Ultra TM RNA library preparation kit (Illumina, New England Biolabs, Ipswich, MA, USA) was used to construct the library. PCR was performed using Phusion High-Fidelity DNA polymerase, Universal PCR primers, and Index Primer. After product purification (AM Pure XP system), the library quality was evaluated using the Agilent Bioanalyzer 2,100 system. Following testing, the library preparations were sequenced on an Illumina platform and paired-end reads were generated. The raw RNA-seq data are available in the NCBI SRA database under the accession number PRJNA663233.

Raw reads in fastq format were first processed using in-house perl scripts. In this step, clean reads were obtained by removing those containing adapters and ploy-N, as well as low-quality reads, from the raw data. At the same time, the Q30, GC-content, and sequence duplication level of the clean data were calculated. The filtered data were compared with the *B. rapa* V3.0 reference genome (http://brassicadb.cn/#/Download/) using HISAT2 (Kim, Langmead & Salzberg, 2015). String Tie (Pertea et al., 2015) was then used to assemble and quantify results. The Fragments Per Kilobase of exon model per Million mapped fragments (FPKM) value of each sample was calculated using Cufflinks V2.2.1 (Trapnell et al., 2012). To evaluate the replicate reproducibility, Pearson correlation analyses were performed in R language.

### Identification and functional annotation of differentially expressed genes

Power analysis calculations were performed on transcriptome samples using the RNASeqPower package (https://doi.org/doi:10.18129/B9.bioc.RNASeqPower) with the following parameter settings: Depth = 20, Effect = 2, and α = 0.05 (Conesa et al., 2016). The DESeq R package (https://bioconductor.org/packages/release/bioc/html/DESeq2.html) was used to analyze the RNA-seq results (Love, Huber & Anders, 2014). Genes with FDR < 0.01 and |log_2_(fold change)|> 2 were identified as differentially expressed in reference to previous methods used to adjust the false discovery rate (FDR) (Benjamini & Hochberg, 1995).

Based on the *B. rapa* V3.0 genome, all DEGs were annotated through the Gene Ontology (GO) and Kyoto Encyclopedia of Genes and Genomes (KEGG) databases to determine their biological functions and participation pathways. GOseq R was used to annotate GO (Young et al., 2010). Gene functions were annotated based on the Swiss-Prot, EuKaryotic Orthologous Groups (KOG)/Clusters of Orthologous Groups (COG), KEGG Orthology, Pfam, and Non-redundant (Nr) NCBI databases.

### Gene temporal expression analysis and hub gene analysis

Through gene co-expression trend analysis, the expression patterns of Chinese cabbage genes under heat stress were studied. We compared DEGs in S268-CK_vs_S334-CK, S268-HT-4_vs_S334-HT-4, S268-HT-8_vs_S334-HT-8, S268-HT-10_vs_S334-HT-10, and S268-RC-4_vs_S334-RC-4 in order to cluster the change patterns of unigenes through the BMKCloud website (www.biocloud.net). DEGs were clustered into 15 expression profiles. Clustered profiles of DEGs with *p* < 0.01 were considered to be significantly different from the reference set for each sample.

Hub genes are genes with the most junctions in each cluster. The *B. rapa* reference genome was analyzed against each cluster’s DEGs to predict the interaction network of these DEGs. Protein interactions were obtained from the STRING database (http://string-db.org/). kME values were used to indicate height. Pearson correlation coefficients between expression levels and module eigengenes were calculated and genetic networks were mapped using Cytoscape software (Chin et al., 2014).

### Fluorescent quantitative real-time PCR

RNA samples were reverse transcribed to cDNA using EasyScript One-Step gDNA Removal and cDNA Synthesis SuperMix Kit (TransGen, Beijing, China) for qPCR. The relative expression level of the DEGs were detected through quantitative real-time PCR using Taq Pro Universal SYBR qPCR Master Mix (Vazyme, Nanjing, China) and CFX-96 Real-time System (BIORAD, Hercules, CA, USA). *Actin* was used as an internal control, and the 2^−ΔΔCT^ method was used to calculate relative gene expression levels (Livak & Schmittgen, 2001). The primer sequences used in the qPCR analysis are shown in Table S13.

### Statistical analyses

GraphPad Prism 8 (San Diego, CA, USA) and Microsoft Office Excel 2010 software (Redmond, WA, USA) packages were used to analyze the data collected in the physiological experiments and by qRT-PCR. IBM SPSS 25.0 (Armonk, NY, USA) tests were used to evaluate the significance of the differences. Tukey’s post-hoc test was used for mean comparisons. *p* < 0.05 was considered statistically significant.

## Results

### Morphological and physiological biochemical changes

**Figure 1:**
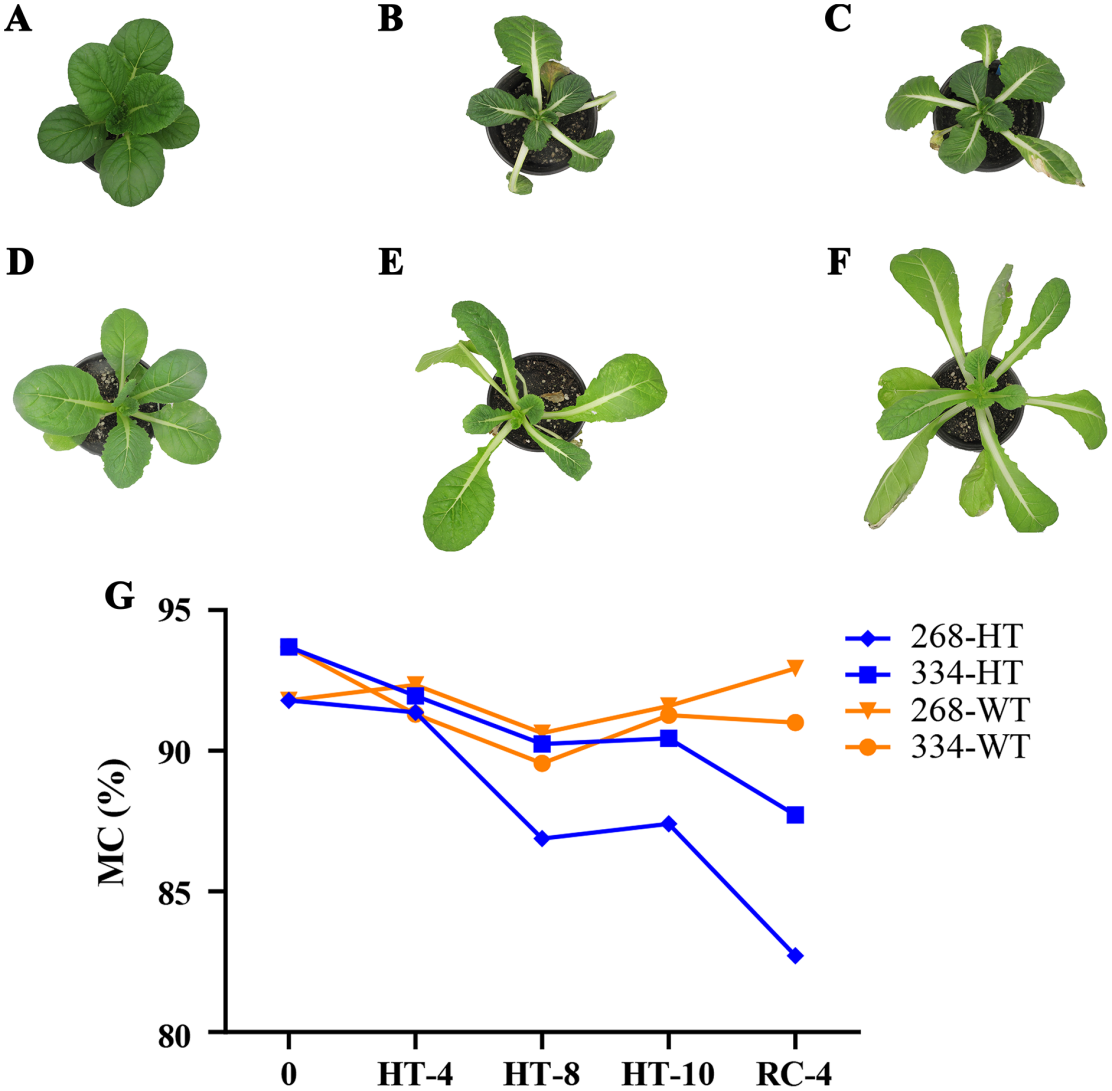
Changes in appearance and moisture content of the ‘268’ and ‘334’ lines. (A–C) Represent the ‘268’ line before heat stress (CK), during heat stress (HT-10) and recovery treatment (RC-4), respectively. (D–F) Represent the ‘334’ line before heat stress (CK), during heat stress (HT-10) and recovery treatment (RC-4), respectively. (G) The moisture content (MC) varied at different treatments.

**Figure 2:**
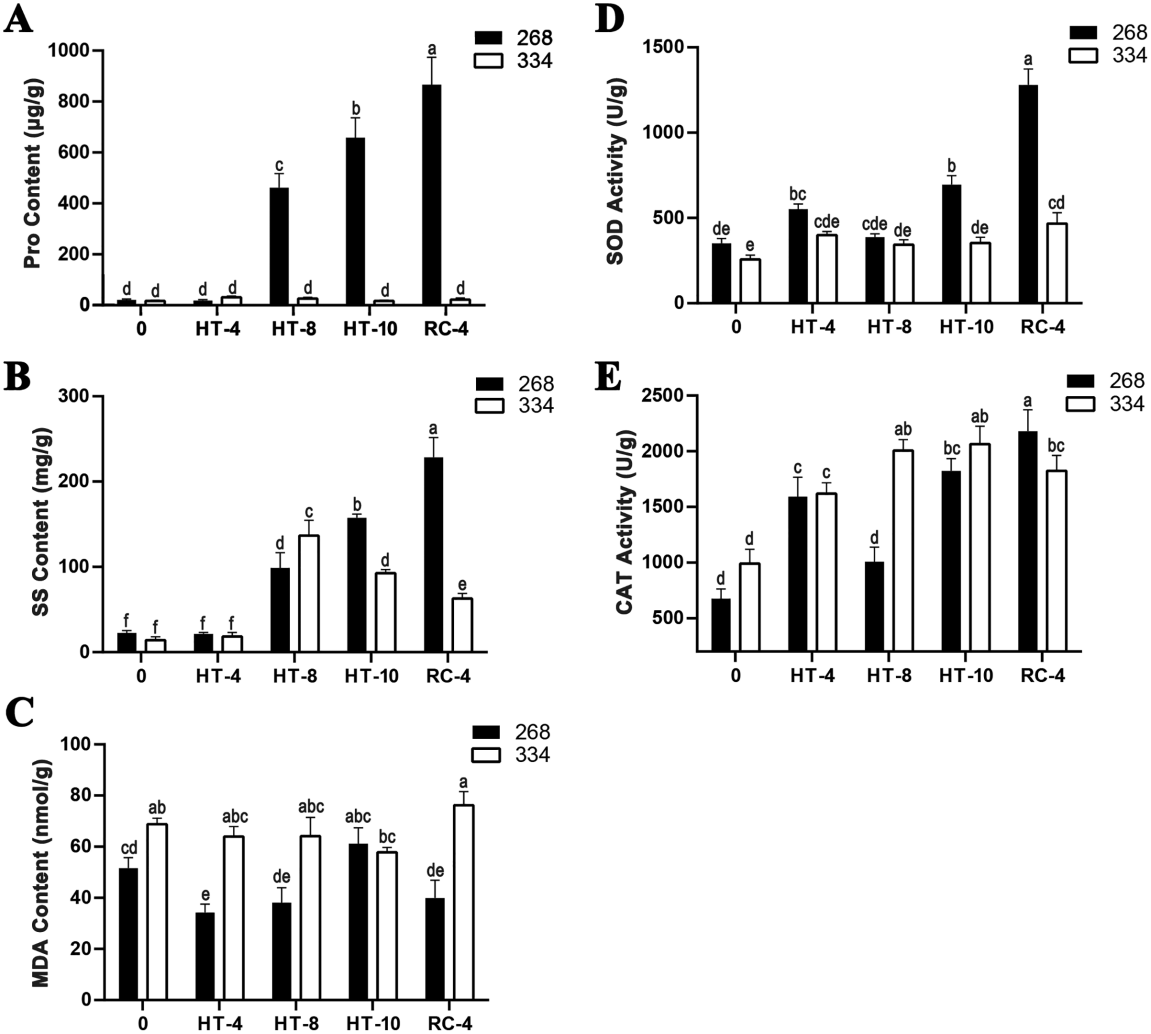
Physiological index measurement. (A) Changes in proline content, (B) soluble sugar content, (C) MDA content, (D) SOD activity, and (E) CAT activity in the leaves of the ‘268’ and ‘334’ lines under heat treatment and recovery. Each data point represents the mean (±SD) of three separate experiments. Tukey’s *post-hoc* test was used for mean comparisons, and different letters indicate significant differences (*p* < 0.05). Error bars represent the SD.

The ‘268’ line had a deeper leaf color, more compact phenotype, and thicker stem than the ‘334’ line (Figs. 1A and 1D). The moisture content (MC) of both lines showed a decreasing trend under heat stress, but the ‘268’ line had a more significant decline, from 91.8% to 82.7% (Fig. 1G). Under heat stress and recovery, the ‘268’ line showed continuous accumulation of Pro and SS content. However, the Pro content of the ‘334’ line changed little across different treatment stages, and the SS content increased first and then decreased (Figs. 2A and 2B). The trend of the MDA content in both lines was very complicated, as reflected by the accumulation level and the time taken to reach the extreme value (Fig. 2C). The SOD activity of the ‘268’ and ‘334’ lines displayed changes similar to that of the Pro content. The difference was that the SOD activity of the ‘268’ line decreased slightly during HT-8 (Fig. 2D). Under heat stress and recovery, the CAT activity of the ‘334’ line increased and was always greater than the activity of the ‘268’ line, but it declined rapidly during RC-4, and the activity level was lower than that of the ‘268’ line (Fig. 2E). Heat stress caused significant damage to the ‘334’ line phenotype, the petioles of all tested plants were too long, and the leaf width became narrow and brittle. After 10 days of heat stress at 40 °C, leaf edge scorching was obvious (Figs. 1E and 1F), but the change in the ‘268’ line was relatively small (Figs. 1B and 1C). This suggests that heat stress may be less damaging to heat-tolerant plants than heat-sensitive plants.

### Transcriptome quality control and DEG analysis

The sequencing results for 30 samples were analyzed, producing 1,522,694,456 raw reads and 211.72 Gb of clean data. Each sample had a minimum of 5.79 Gb of clean data, a Q30 percentage of 94.18% or more, and a comparison efficiency ranging from 86.85% to 92.66%. After the removal of rRNA, an average of 90.52% reads were mapped to the reference database (http://brassicadb.cn) (Table 1). The mapped files were then processed by HISAT2 and String Tie to generate a consensus transcriptome assembly containing 44,161 known and 3,529 novel genes.

**Table 1:** Transcriptome data quality and mapping information.

Samples	Samples description	Total reads	Clean bases	GC Content (%)	Clean reads	% ≥ Q30	Mapped reads
S268CA001	268 CK 1	44,955,896	6,698,062,230	48.76	22,477,948	94.10	40,234,675 (89.50%)
S268CA002	268 CK 2	64,495,680	9,610,220,600	49.13	32,247,840	94.71	59,763,167 (92.66%)
S268CA003	268 CK 3	51,913,620	7,754,169,994	49.02	25,956,810	94.84	47,864,430 (92.20%)
S268MA201	268 HT-4 1	48,313,626	7,204,546,114	48.56	24,156,813	94.87	44,465,876 (92.04%)
S268MA202	268 HT-4 2	47,104,864	7,032,568,492	48.46	23,552,432	94.61	43,316,424 (91.96%)
S268MA203	268 HT-4 3	47,976,302	7,153,744,322	48.09	23,988,151	94.70	43,474,359 (90.62%)
S268MA401	268 HT-8 1	43,155,214	6,455,065,634	47.35	21,577,607	94.57	38,764,054 (89.82%)
S268MA402	268 HT-8 2	65,925,896	9,839,072,470	47.80	32,962,948	94.68	59,967,484 (90.96%)
S268MA403	268 HT-8 3	46,370,344	6,934,916,466	46.85	23,185,172	94.18	41,233,551 (88.92%)
S268MA501	268 HT-10 1	61,225,388	9,128,224,528	47.89	30,612,694	94.54	55,531,718 (90.70%)
S268MA502	268 HT-10 2	48,168,448	7,191,594,340	47.46	24,084,224	94.64	43,488,907 (90.29%)
S268MA503	268 HT-10 3	44,840,998	6,701,917,934	47.51	22,420,499	94.44	40,101,321 (89.43%)
S268MA701	268 RC-4 1	59,872,374	8,947,019,214	47.98	29,936,187	94.59	54,549,200 (91.11%)
S268MA702	268 RC-4 2	58,677,732	8,756,353,364	48.28	29,338,866	94.67	53,020,908 (90.36%)
S268MA703	268 RC-4 3	56,337,760	8,418,355,258	48.15	28,168,880	94.50	51,234,044 (90.94%)
S334CA001	334 CK 1	47,355,436	7,064,862,754	47.87	23,677,718	94.72	42,922,082 (90.64%)
S334CA002	334 CK 2	47,297,882	7,055,391,182	48.26	23,648,941	94.87	43,375,482 (91.71%)
S334CA003	334 CK 3	48,678,662	7,280,384,682	47.62	24,339,331	94.30	44,362,183 (91.13%)
S334MA201	334 HT-4 1	44,896,764	6,710,137,738	48.13	22,448,382	94.46	41,006,028 (91.33%)
S334MA202	334 HT-4 2	55,928,874	8,345,476,404	48.49	27,964,437	94.76	51,160,272 (91.47%)
S334MA203	334 HT-4 3	52,388,724	7,817,170,530	48.26	26,194,362	94.71	47,864,510 (91.36%)
S334MA401	334 HT-8 1	42,708,832	6,378,631,492	47.40	21,354,416	94.64	37,842,547 (88.61%)
S334MA402	334 HT-8 2	46,841,358	6,999,312,260	46.94	23,420,679	94.34	40,681,498 (86.85%)
S334MA403	334 HT-8 3	45,440,244	6,780,426,808	47.88	22,720,122	95.09	41,426,280 (91.17%)
S334MA501	334 HT-10 1	45,916,176	6,855,957,602	46.45	22,958,088	94.22	40,299,123 (87.77%)
S334MA502	334 HT-10 2	53,514,198	7,995,907,342	47.49	26,757,099	94.68	48,196,566 (90.06%)
S334MA503	334 HT-10 3	41,644,436	6,214,086,824	47.33	20,822,218	94.78	37,731,389 (90.60%)
S334MA701	334 RC-4 1	55,337,188	8,262,647,300	46.82	27,668,594	94.48	49,537,236 (89.52%)
S334MA702	334 RC-4 2	52,038,402	7,771,459,604	48.02	26,019,201	94.72	47,321,758 (90.94%)
S334MA703	334 RC-4 3	53,373,138	7,975,146,584	47.99	26,686,569	94.47	48,455,715 (90.79%)

To ensure sufficient depth and biological replication of the RNA-seq data, we used RNASeqPower to calculate the statistical power, which was greater than 0.92 for each group of samples (Table S1). Pearson’s correlation coefficients were used to test for biologically repeated correlations between samples. The generated cluster heatmap was used to observe the overall transcriptome correlation of the ‘268’ and ‘334’ lines under different treatments (Fig. S1). The results showed that the three biological replicates from each time period and the transcriptome data both exhibited good correlation.

**Figure 3:**
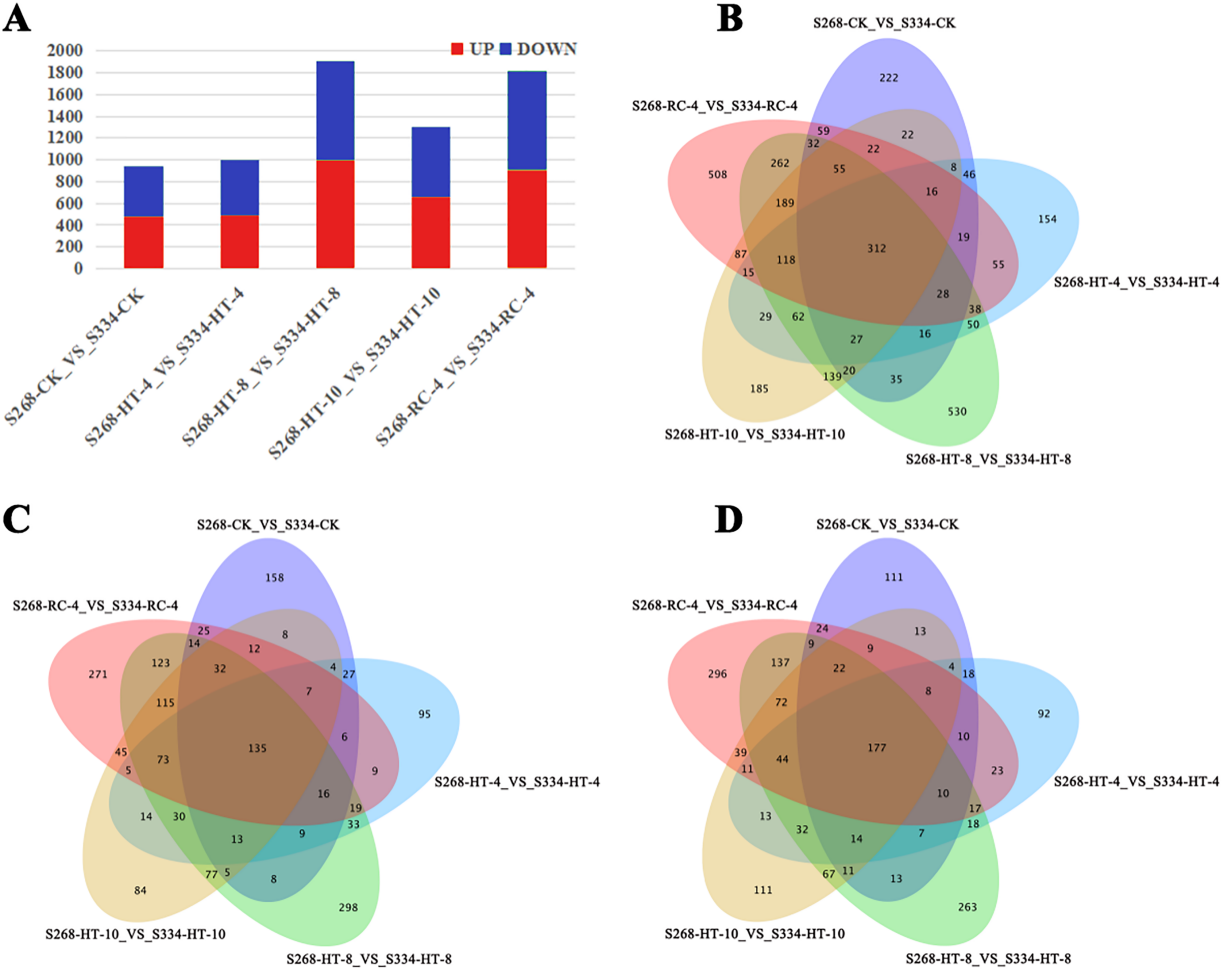
Venn diagrams of DEGs in the two kinds of Chinese cabbage under different heat stress. (A) DEG numbers of the ‘268’ and ‘334’ lines related to the thermal response in the same period. Orange represents upregulated genes, and green represents downregulated genes. (B–D) Venn diagrams of DEGs between the ‘268’ and ‘334’ lines. (B) All DEGs; (C) upregulated DEGs; (D) downregulated DEGs. FDR < 0.01 and |log_2_(fold change)|> 2.

Gene expression was analyzed based on FPKM, the ‘334’ line under the same treatment was used as a control, and the transcriptional results of the ‘268’ line were analyzed to identify DEGs. In different treatment groups, S268-CK_vs_S334-CK and S268-HT-4_vs_S334-HT-4 had the lowest number of DEGs, while S268-HT-8_VS_S334-HT-8 had the most significant difference (Fig. 3A). Comparisons of these five datasets showed that 118 genes overlapped among HT-4, HT-8, HT-10, and RC-4. Among these, 73 genes were upregulated and 44 genes were downregulated. These genes may play crucial roles in the heat-tolerance of Chinese cabbage (Figs. 3B–3D; Tables S2 and S3).

### GO and KEGG analyses of DEGs

**Figure 4:**
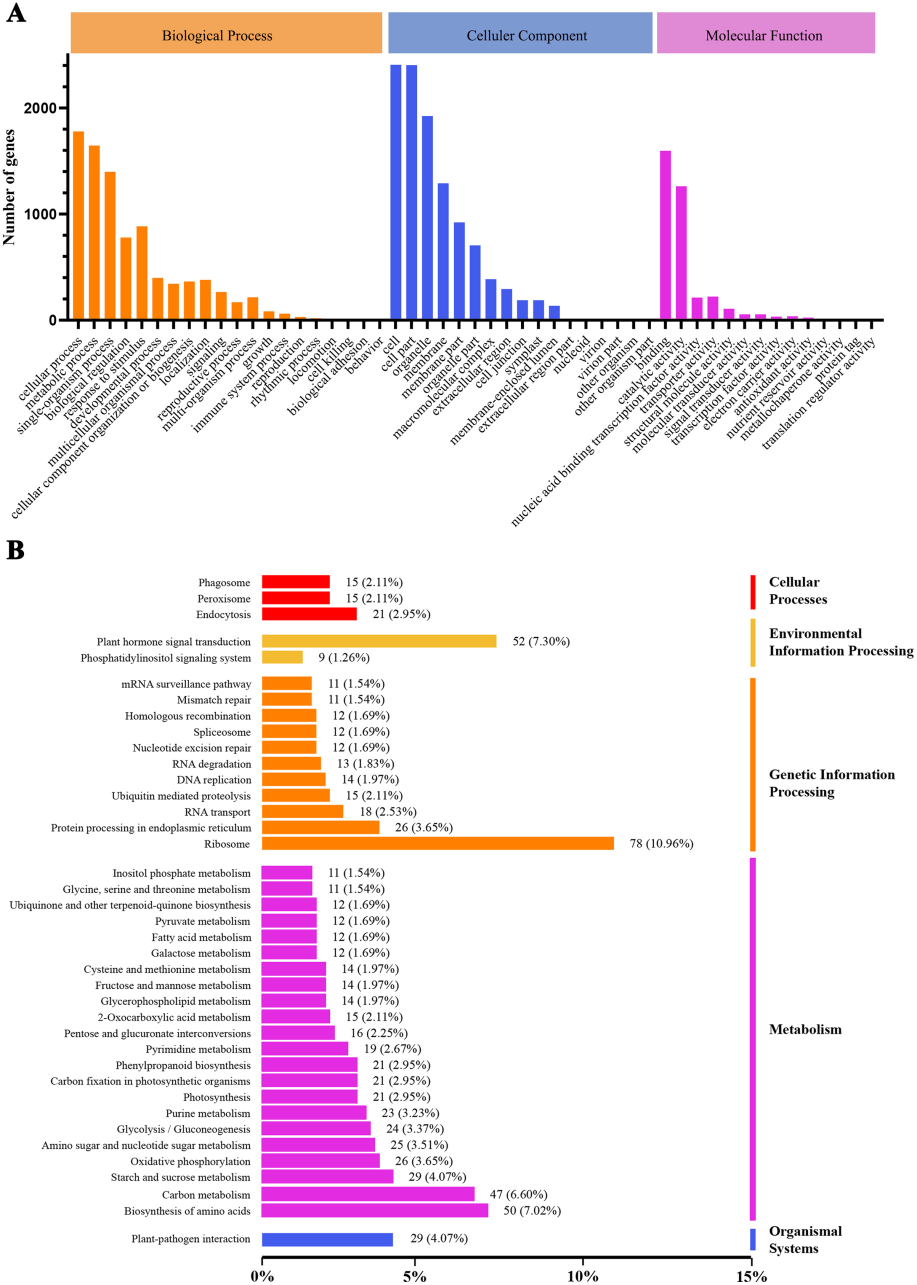
GO classification and KEGG enrichment analysis of DEGs. (A) GO classification of DEGs; (B) KEGG classification of DEGs.

We conducted GO and KEGG pathway analyses of the DEGs. A total of 2,815 GO terms were assigned to the 3,360 DEGs that responded to heat stress (Fig. 4A). Pathways with higher enrichment were screened in KEGG and formed five main categories and 39 subcategories (Fig. 4B). The most abundant category was ‘metabolism’, distributed over a large number of subcategories, followed by ‘genetic information processing’. In ‘metabolism’, the most abundant subcategories were ‘biosynthesis of amino acids’, ‘carbon metabolism’, and ‘starch and sucrose metabolism’ with enrichment levels of 50, 47, and 29 DEGs, respectively. The richest subcategory in ‘genetic information processing’ was ‘ribosome’ with 78 (10.96%) DEGs. ‘Plant hormone signal transduction’ was also an enriched subcategory with 52 (7.30%) DEGs.

### Dynamic gene expression pattern analyses during heat stress

**Figure 5:**
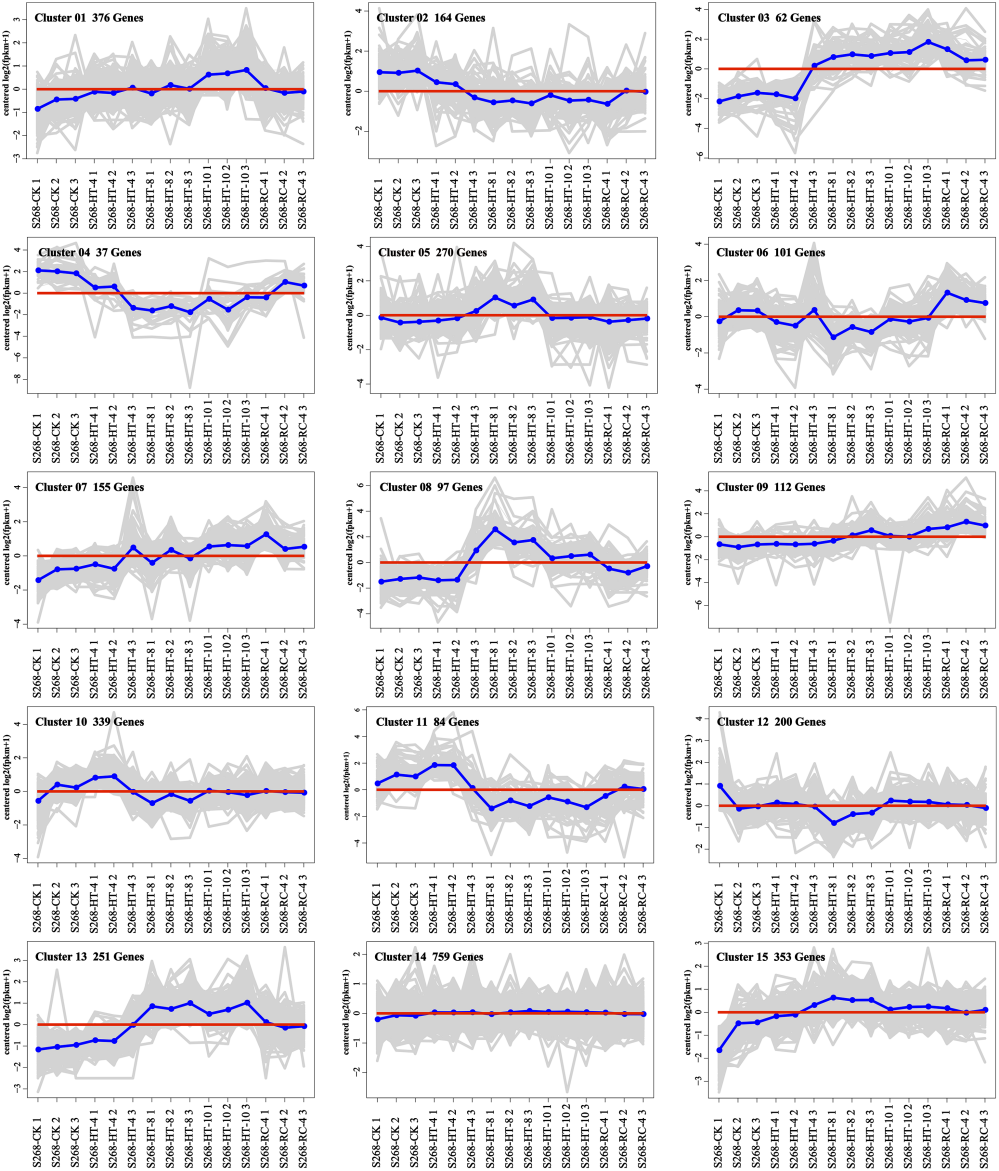
Expression clusters of DEGs under heat stress and recovery conditions. Each square represents one cluster. The *x*-axis represents the treatment time, and the *y*-axis represents expression.

**Figure 6:**
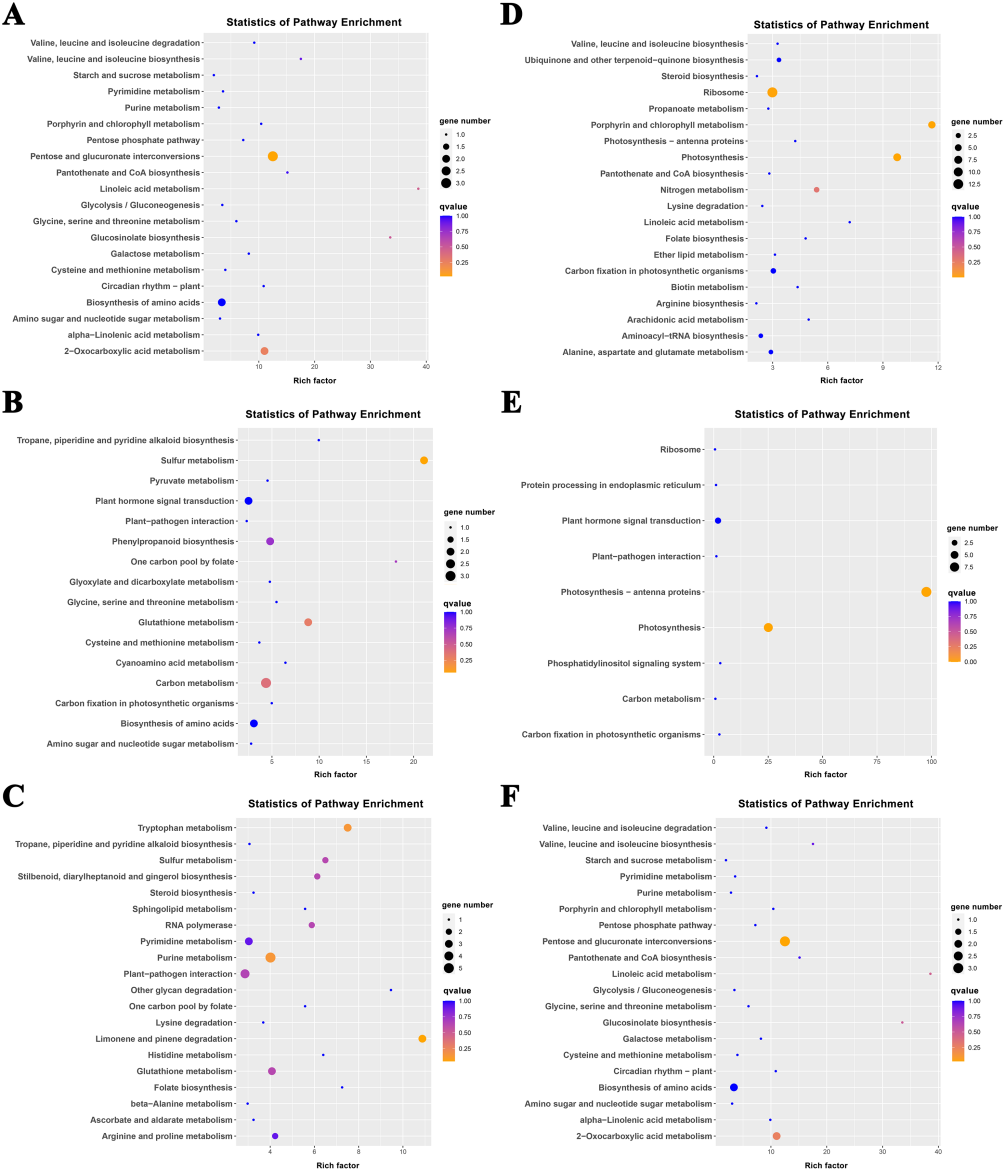
KEGG pathway enrichment of Clusters (A) 1, (B) 3, (C) 13, (D) 2, (E) 4, and (F) 11. The size of the circle represents the number of genes enriched in the pathway, and the depth of the color represents the size of the *q* value.

The K-means method was used to analyze the dynamic changes of gene expression in the ‘268’ line, and all DEGs were classified into 15 clusters with similar expression profiles (Fig. 5). In Clusters 1, 3, and 13, the expression level of enriched DEGs showed a gradual induction state under heat stress, and a decrease during RC-4. The expression trends of DEGs in the enriched Clusters 2, 4, and 11 continued to decrease under heat stress and increased during RC-4. GO and KEGG analyses indicated that ‘carbon metabolism’, ‘biosynthesis of amino acids’, ‘plant hormone signal transduction’, ‘purine metabolism’, ‘glutathione metabolism’, and ‘plant–pathogen interaction’ were jointly enriched in Clusters 1, 3, and 13 (Figs. 6A–6C; Table S4). Many genes in Clusters 2, 4 and 11 were classified together into ‘photosynthesis’, ‘photosynthesis-antenna proteins’, ‘ribosome’, ‘porphyrin and chlorophyll metabolism’, ‘carbon metabolism’, ‘plant hormone signal transduction’, and ‘biosynthesis of amino acids’. It should be noted that, in Cluster 4, the ‘photosynthesis-antenna proteins’ (nine genes) and ‘photosynthesis’ (seven genes) pathways were significantly enriched. (Figs. 6D–6F; Table S5). The results suggest that these genes may be closely related to heat stress.

Clusters 8 and 15 showed increased gene expression during heat stress and recovery compared with the S268-CK with the greatest differential expressions both occurring during HT-8 (Fig. 5). The KEGG enrichment pathway of Cluster 8 indicated extensive involvement of these genes in the synthesis and degradation of amino acids (glutamate, alanine, and aspartate), the degradation and metabolism of fatty acids, and the biosynthesis of various secondary metabolites (Table S6). Notably, Cluster 8 underwent a decrease in gene expression levels during RC-4 with little difference when compared with the S268-CK. Combined with KEGG annotations, Cluster 15 was enriched for a large number of genes in ‘starch and sucrose metabolism’ (ko00500), ‘glycolysis/gluconeogenesis’ (ko00010), ‘Galactose metabolism’ (ko00052), ‘fructose and mannose metabolism’ (ko00051), and ‘amino sugar and nucleotide sugar metabolism’ (ko00520) (Table S6), including the BraA06g003260.3C. gene (Hexokinase-Like 1, *HKL1*), BraA01g028060.3C. gene (*TPS10*), BraA03g011780.3C. gene (*BGLU4*), BraA05g040820.3C. gene (*SS2*), BraA01g001150.3C. gene (*PCKA*), and BraA10g021910.3C. gene (*At5g18200*). The *HKL1*, *TPS10*, *BGLU4*, and *SS2* genes play important roles in starch and sucrose metabolism. Additionally, many sugar metabolism pathways were also enriched in Cluster 1. This indicates that the expression of genes related to sugar metabolism in the heat-tolerant ‘268’ line was generally upregulated under heat stress (Table S4).

**Figure 7:**
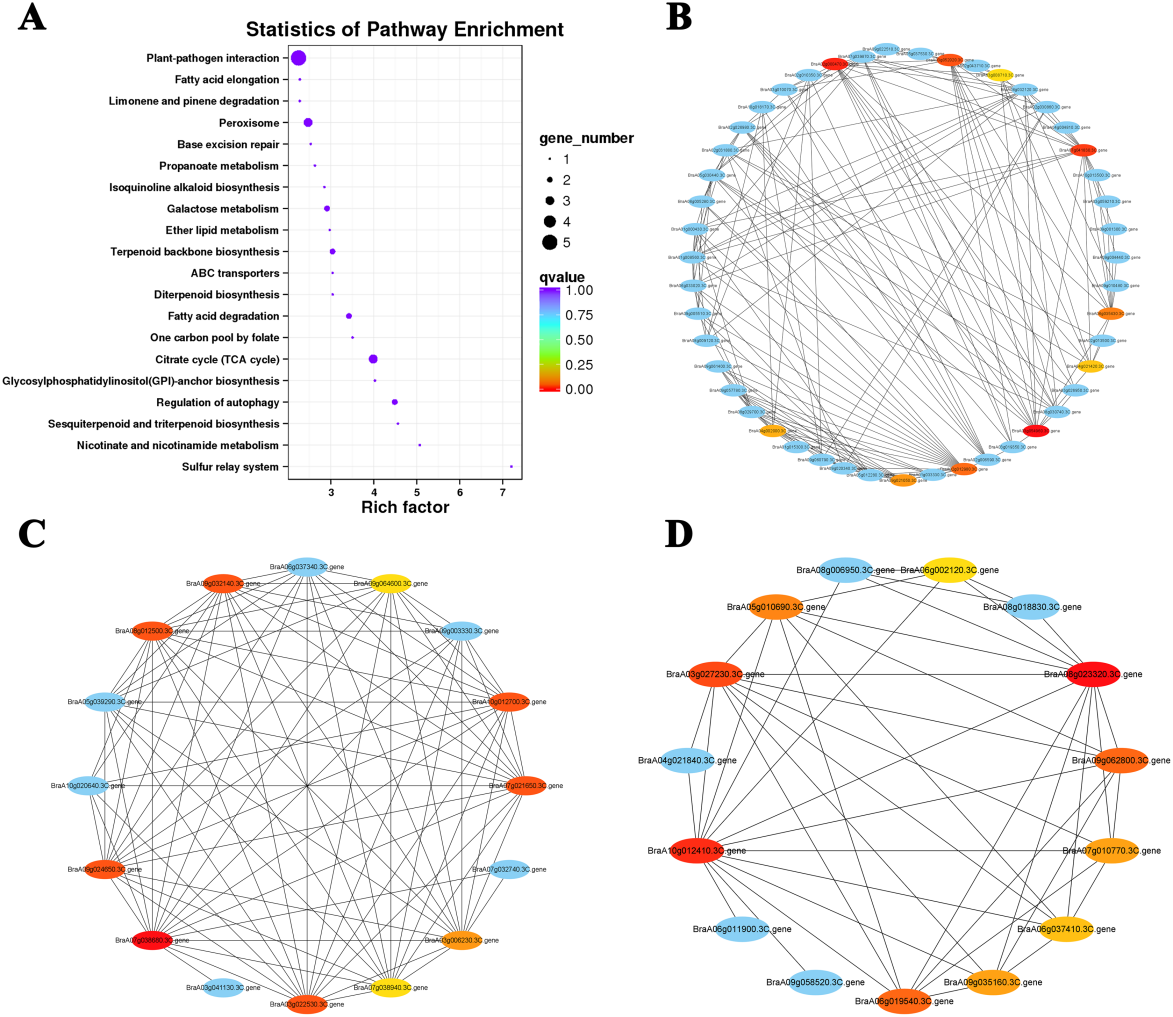
KEGG pathway enrichment and putative interaction networks of clusters. (A) KEGG pathway enrichment of Cluster 15, (B) putative interaction networks of Cluster 15, (C) putative interaction networks of Cluster 2, (D) putative interaction networks of Cluster 4. Colors from red to orange to yellow represent the top 10 hub genes with strong to weak correlations, and blue represents other genes associated with hub genes. The size of the circle represents the number of genes enriched in the pathway, and the depth of the color represents the *q* value.

The top 10 hub genes of Cluster 15 were screened with the DEG interaction network for analysis (Figs. 7A and 7B; Table S7). The results showed that five hub genes were associated with ABA levels: *BraA03g054960.3C. gene* (*TZF3*), *BraA02g000470.3C. gene* (*RPT6*), *BraA03g052020.3C. gene* (*NBR1*), *BraA06g035430.3C. gene* (*STUBL1*), and *BraA03g008710.3C. gene* (*MIEL1*). An interaction network analysis was also performed on the downregulated Cluster 4 (Fig. 7D). We found that all 10 hub genes clustered in the ‘photosynthesis’ (ko00195) and ‘photosynthesis-antennal protein’ (ko00196) pathways (Table 2). Six of the seven genes associated with the light-harvesting chlorophyll a/b-binding (*LHC*) gene family belong to the *LHCB* gene subfamily (*LHCB3*, *LHCB5*, *LHCB1.4*, *LHCB1.3*, and *LHCB2.4*) and one belongs to the *LHCA* gene subfamily (*LHCA4*). Under heat stress and recovery, these genes showed a downward trend in both lines, but the expression levels in the ‘268’ line were always higher than those in the ‘334’ line during the same period. This indicates that the photosystem may be destroyed under heat stress with a significant reduction in photosynthetic efficiency, which is consistent with the observed plant phenotype.

**Table 2:** Hub gene annotation of Cluster 4.

Gene ID	Gene name	Description	KEGG_pathway
BraA08g023320.3C. gene	*PSAK*	Photosystem I reaction center subunit psaK, chloroplastic	Photosynthesis (ko00195)
BraA10g012410.3C. gene	*LHCB3*	Chlorophyll a-b binding protein 3, chloroplastic	Photosynthesis—antenna proteins (ko00196)
BraA03g027230.3C. gene	*LHCB5*	Chlorophyll a-b binding protein CP26, chloroplastic	Photosynthesis—antenna proteins (ko00196)
BraA06g019540.3C. gene	*LHCA4*	Chlorophyll a-b binding protein 4, chloroplastic	Photosynthesis—antenna proteins (ko00196)
BraA09g062800.3C. gene	*PSAO*	Photosystem I subunit O	Photosynthesis (ko00195)
BraA05g010690.3C. gene	*LHCB1.4*	Chlorophyll a-b binding protein 1, chloroplastic	Photosynthesis—antenna proteins (ko00196)
BraA07g010770.3C. gene	*LHCB1.3*	Chlorophyll a-b binding protein 1, chloroplastic	Photosynthesis—antenna proteins (ko00196)
BraA09g035160.3C. gene	*LHCB1.3*	Chlorophyll a-b binding protein 1, chloroplastic	Photosynthesis—antenna proteins (ko00196)
BraA06g037410.3C. gene	*LHCB2.4*	Chlorophyll a-b binding protein 2.4, chloroplastic	Photosynthesis—antenna proteins (ko00196)
BraA06g002120.3C. gene	*PSAH*	Photosystem I reaction center subunit VI, chloroplastic	Photosynthesis (ko00195)

The interaction networks of DEGs were screened in Clusters 1 and 2, and the top 10 hub genes for both selected clusters were associated with ribosomal proteins (Fig. 7C; Tables S8 and S9). The difference was that genes related to 80S ribosomal proteins were significantly enriched in Cluster 1. In contrast, Cluster 2 was enriched for many genes related to 70S ribosomal protein, which is widely involved in the composition of chloroplast ribosomes (Ahmed, Shi & Bhushan, 2017). Under heat stress, the level of the chloroplast ribosome-encoded protein was weakened, and the expression of some light energy capture factors was suppressed, which may have led to a decrease in photosynthesis intensity.

### Identification of ROS-scavenger and heat shock transcription factor genes

**Figure 8:**
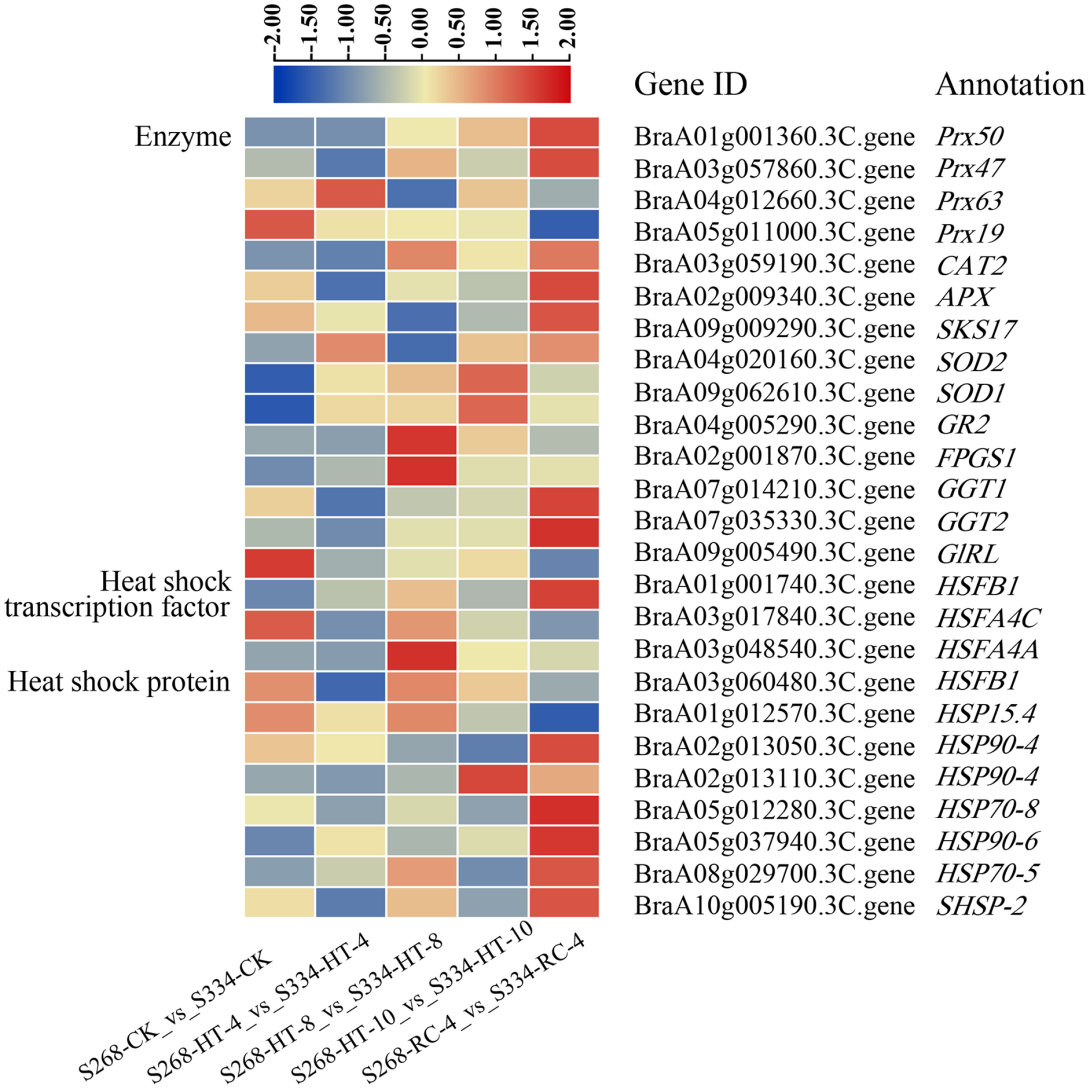
DEGs related to enzymes, heat shock transcription factors, and heat shock proteins under different heat stress conditions. The heatmap was generated using TBtools software, and the five boxes in each horizontal row correspond to five treatments.

To elucidate the molecular mechanisms of the ‘268’ and ‘334’ lines under heat stress, 18 enzymes, five HSFs, and 14 HSP-related genes were identified in all DEGs (Fig. 8; Tables S10–S12). The *BraA01g001360.3C. gene* (Peroxidase 50, *Prx50*), *BraA10g028260.3C. gene* (Peroxidase 52, *Prx52*), *BraA10g029420.3C. gene* (Peroxidase 54, *Prx54*), and *BraA10g006200.3C. gene* (Phospholipid hydroperoxide glutathione peroxidase 1, *GPX1*) showed significantly higher expression levels in the ‘268’ line than in ‘334’ line. The expression levels of the *BraA09g062610.3C. gene* (Superoxide dismutase, *SOD1*) and *BraA04g020160.3C. gene* (Superoxide dismutase 2, *SOD2*) in the ‘268’ line increased continuously compared with the ‘334’ line, but the levels decreased during RC-4, indicating that the two genes were more responsive to heat stress (Fig. 8, Table S10). During treatment, the *BraA04g028620.3C. gene* (Heat stress transcription factor A-1, *HSFA1*) was expressed only in the ‘268’ line, and the expression level gradually increased with the treatment time and then returned to the CK level during RC-4. Activation of HSPs reduces the likelihood of protein denaturation and improves the stress capacity of an organism. Two new genes, *Brassica_rapa_newGene_7058* and *Brassica_rapa_newGene_7942*, were identified. Before treatment, the expression levels of the two genes were similar in the ‘268’ and ‘334’ lines, but the expression levels of the ‘268’ line increased significantly under heat stress and suddenly decreased during RC-4. In contrast, the gene expression in the ‘334’ line didn’t change under different treatment. We speculated that these two genes likely play active roles in heat stress in the heat-tolerant ‘268’ line (Fig. 8, Tables S11 and S12).

### The DEGs from the transcriptome analysis detected by qRT-PCR

**Figure 9:**
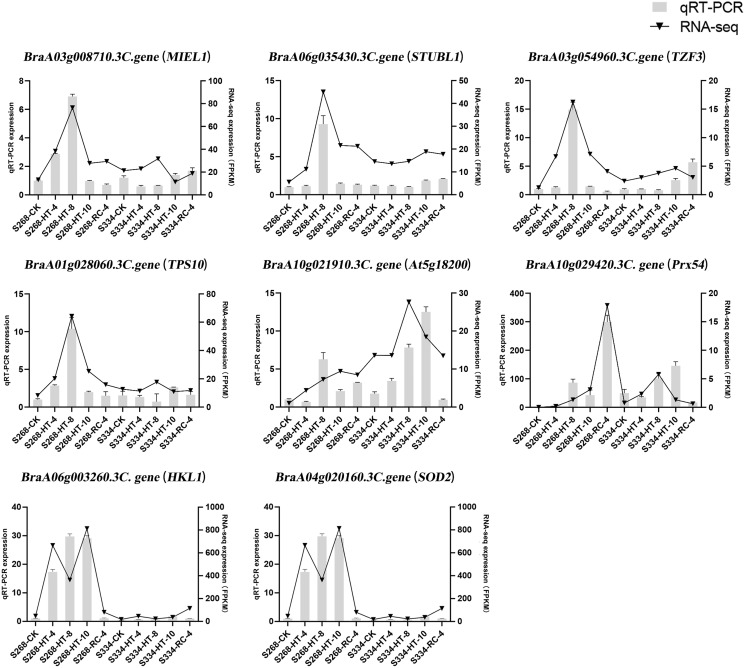
Relative expression of eight selected DEGs analyzed by qRT-PCR and RNA-seq expression trends. Bar and line graphs represent the qRTPCR and RNA-seq data, respectively. Data are presented as the mean standard error (SE).

To confirm the results of the RNA-seq analysis, eight DEGs were verified using qRTPCR in *B. rapa*, including three genes related to ABA levels (*MIEL1*, *STUBL1*, and *TZF3*), three genes related to sugar metabolism (*HKL1*, *TPS10*, and *At5g18200*), and two genes related to ROS-scavenger activity (*Prx54* and *SOD2*) (Fig. 9). The RNA-seq analysis and the qRT-PCR results were consistent across time periods, indicating the reliability of transcriptome sequencing. The expression level of ROS scavenger-related genes in the ‘268’ line was higher than that of the ‘334’ line, which was the same as the RNA-seq data, suggesting that these genes may be involved in plant stress tolerance.

## Discussion

In this study, heat stress experiments were performed on heat-tolerant and heat-sensitive Chinese cabbage lines through combined analyses of transcriptome and physiological indicators. We identified 3,360 DEGs using RNA-seq analysis. Next, using the measured physiological indicators combined with the functional annotation of DEGs, we discovered many metabolic pathways related to heat stress as well as some key genes that may be involved in the thermal response.

To avoid injury due to heat stress, plants have developed a series of strategies (Chen & Yang, 2019). The accumulation of SS can significantly improve the stress tolerance of plants (Sami et al., 2016). Pro is an essential amino acid that maintains the plant’s subcellular structure and participates in antioxidant defense systems. In this study, changes in Pro and SS content in the ‘268’ and ‘334’ lines under heat stress and recovery (Figs. 2A and 2B) were consistent with those found in previous studies (Kim et al., 2013). Furthermore, higher SS content had a positive effect on the accumulation of Pro and the regulation of ROS catabolism (Couee et al., 2006; Hu et al., 2012). Similar results were observed in our experiments, where the SOD activity of the ‘268’ line was consistently higher than that of the ‘334’ line (Fig. 2D). Under heat stress, SS and Pro act as osmoregulatory substances *in vivo*, protecting organelles, maintaining membrane system stability, and helping plants establish osmotic homeostasis (Hasanuzzaman et al., 2013; Rosa et al., 2009; Xue, Liu & Hua, 2009; Yu et al., 2021). Interestingly, heat stress significantly increased the Pro and SS content of the ‘268’ line, but the content did not return to normal after recovery treatment. This shared some similarities with previous research. After recovery treatment in *Freesia* seedlings, the Pro content decreased, but the SS content remained unchanged (Yuan et al., 2011). We speculate that on one hand, plants regulate physiological and biochemical responses *in vivo* through thermal acclimation, and this ability can be maintained for days or even longer, though returning to normal temperatures, and this is mainly reflected in the high expression of heat stress-related genes and the accumulation of osmotic regulators (Su et al., 2021). On the other hand, long-term heat stress may destroy the normal growth and material metabolism of plants so that the heat stress response mechanism cannot be recovered. This speculation may also be responsible for the specificity of SOD activity (Fig. 2D). In short, when compared with the ‘334’ line, the continuous accumulation of SS and Pro in the ‘268’ line strengthens osmotic regulation and ROS metabolism, which help to slow down the damage to cell tissues caused by high temperatures and make plants more heat-tolerant.

When plants are exposed to high temperatures for long periods of time, the aerobic metabolism of organelles, such as the mitochondria, chloroplasts, and peroxisomes, increases, leading to the accumulation of ROS and damage to DNA, proteins, and membrane systems. This results in decreased cell function and weakened plant adaptability (Bokszczanin et al., 2013; del Rio et al., 2006; Navrot et al., 2007). To restore redox homeostasis and cellular damage, plants activate ROS scavenging mechanisms. Previous studies have shown that the induction of ROS scavenging genes in heat-tolerant plants is stronger than in heat-sensitive plants (Almeselmani et al., 2006; Gupta et al., 1993b; Wu et al., 2012). In our study, the ‘268’ line showed gradually increased SOD and CAT activities under heat stress and recovery, and decreased slightly during the HT-8 period. The SOD activity of the ‘334’ line was always maintained at a low level with no significant changes, while the CAT activity continued to increase under heat stress and was always higher than that of the ‘268’ line. However, during RC-4, it decreased rapidly and was lower than that of the ‘268’ line. Interestingly, the MDA content of the ‘334’ line decreased slowly with heat stress, but accumulated rapidly during RC-4. The MDA content of the ‘268’ line decreased more significantly compared with the CK period, and only abnormally accumulated during HT-10 (Figs. 2C–2E). These results were similar to previous observations of antioxidant enzyme activity in *Brassica oleracea* under heat stress treatment (Soengas et al., 2018). Contrary to this result, high temperature treatment (35 °C/30 °C) of canola seedlings resulted in a significant decrease in SOD activity and an increase in CAT activity (Zhang et al., 2015). This shows that the response of antioxidant enzymes to temperature is not invariant and is highly dependent on the plant organ, species, growth stage, exposure time, and temperature (Almeselmani et al., 2006; Lee et al., 2004; Lu, Sang & Ma, 2008; Toivonen & Sweeney, 1998). This also shows that the use of POD and CAT activity to identify heat-tolerance in Chinese cabbage needs to be confirmed by further research.

SS are crucial in plants and can be used as physiological indicators to identify plant tolerance levels. In combination with Cluster 15, sugar metabolism-related pathways are enriched with many genes, including *HKL1*, *TPS10*, *BGLU4*, *SS2*, *PCKA*, and At5g18200 (Table S6). *HKL1* is an important member of the hexokinase family that has sugar signal transduction functions (Pego, Weisbeek & Smeekens, 1999). Recent studies have shown that *AtHKL1* is a negatively-regulated gene in *Arabidopsis*, and that overexpression of *AtHKL1* reduces glucose binding affinity, which slows down glucose catabolism so that it is available for adversity tolerance (Karve & Moore, 2009). Under elevated Glc conditions, *HKL1* acts as a regulatory protein that may promote ethylene accumulation and attenuate plant growth by inhibiting the activity of the *Arabidopsis* E8 homologous protein (Karve, Xia & Moore, 2012). In this study, the ‘268’ and ‘334’ lines had similar *HKL1* gene expression levels before heat stress, but as the treatment time increased, the gene expression level of the ‘268’ line was significantly higher than that of the ‘334’ line. Along with the accumulation of glucose content, *HKL1* may play an important role in regulating heat tolerance.

The application of exogenous plant hormones significantly ameliorates heat damage in plants, suggesting that plant hormones are involved in the heat stress response (Li et al., 2020). In the interaction network of Cluster 15, five of the top 10 hub genes were found to be associated with phytohormones, with four of these able to be regulated in response to ABA (Figs. 7A and 7B; Table S7). ABA is a kind of isoprene (Nambara & Marion-Poll, 2005), which is one of the most important plant hormones involved in plant growth, development, and adaptation to various stress conditions (Merlot et al., 2001). The transcript levels of these five ABA-related genes continued to accumulate under heat stress and recovery treatments. Among them, the positive regulators of ABA, *BraA03g054960.3C. gene* (*OZF2*), *BraA02g000470.3Cgene* (*PRT6*), and *BraA03g052020.3C. gene* (*NBR1*), showed more significant increases in transcription levels in the ‘268’ line under heat stress, which was consistent with previous studies (Castillo et al., 2021; Lamichhane et al., 2020; Lee et al., 2012; Tarnowski et al., 2020; Thirumalaikumar et al., 2021). In contrast, the *BraA03g008710.3C. gene* (*MIEL1*), a negative regulator gene of ABA (Lee & Seo, 2016), also showed significantly higher expression in the ‘268’ line, and we consider that it may play a role in balancing ABA levels *in vivo*. These changes also suggest that ABA signaling is an important mechanism in the response to abiotic stresses.

All DEGs were analyzed, and a total of 18 ROS scavenger enzyme-related genes were annotated (Fig. 8, Table S10), including seven *Prx* (peroxidase) genes, two *SOD* (superoxide dismutase) genes, and a *CAT* (catalase) gene. *Prxs* is a plant-specific gene that plays a key role in plant resistance to abiotic stress (Broin et al., 2002; Konig et al., 2002; Vidigal et al., 2013; Xie et al., 2021). SOD is an important part of a plant’s active oxygen scavenging system, as it can quickly convert superoxide into hydrogen peroxide (H_2_O_2_) and molecular oxygen, which form the first barrier against superoxide free radicals (Fridovich, 1995; Gupta et al., 1993a). In this study, the expression levels of *SOD1* and *SOD2* genes in the ‘268’ line were similar to those in the ‘334’ line prior to heat stress, but during high-temperature treatment, the expression levels of both genes were significantly higher in the ‘268’ line, and they decreased again during the recovery treatment. The change in *SOD2* was more significant than the change in *SOD1*. While high temperatures caused the accumulation of ROS *in vivo*, superoxide-dismutase-related genes were first activated to promote the elevation of superoxide dismutase activity, which effectively scavenged the generated ROS and reduced the damage suffered by cells. Furthermore, other ROS scavenging enzymes, such as peroxidase, ascorbate peroxidase, and catalase, showed significant changes in the expression levels of genes related to these enzymes, which together constitute the plant’s active oxygen scavenging system.

## Conclusion

In this study, we performed a comprehensive comparative transcriptome analysis of the heat-sensitive and heat-tolerant Chinese cabbage lines in order to identify their gene expression and analyze their molecular mechanisms. This study revealed that under heat stress and recovery treatment, the unigenes of plant hormone signal transduction, amino acid biosynthesis, carbon metabolism, and glycolysis/gluconeogenesis played important roles in the heat-tolerance of the two lines. Additionally, when combined with physiological indicators, the hub genes that were uniquely expressed in the heat-tolerant line and closely related to heat stress were identified. Ultimately, these findings provide us with a deeper understanding of the molecular mechanisms of heat stress and could facilitate the genetic improvement of heat tolerance in Chinese cabbage.

## Supplemental Information

10.7717/peerj.13427/supp-1 Supplemental Information 1 Heatmap of correlation value (R square) of 30 libraries. The color scale (red to blue) indicates correlation between samples

10.7717/peerj.13427/supp-2 Supplemental Information 2 Statistical power of transcriptome sample size.

10.7717/peerj.13427/supp-3 Supplemental Information 3 Genes up-regulated in HT-4, HT-8, HT-10 and RC-4.

10.7717/peerj.13427/supp-4 Supplemental Information 4 Genes down-regulated in HT-4, HT-8, HT-10 and RC-4.

10.7717/peerj.13427/supp-5 Supplemental Information 5 List of up-regulated KEGG pathway Clusters.

10.7717/peerj.13427/supp-6 Supplemental Information 6 List of down-regulated KEGG pathway Clusters.

10.7717/peerj.13427/supp-7 Supplemental Information 7 List of Cluster 8 and 15 KEGG pathway.

10.7717/peerj.13427/supp-8 Supplemental Information 8 Hub gene annotation of Cluster 15.

10.7717/peerj.13427/supp-9 Supplemental Information 9 Hub gene annotation of cluster 1.

10.7717/peerj.13427/supp-10 Supplemental Information 10 Hub gene annotation of cluster 2.

10.7717/peerj.13427/supp-11 Supplemental Information 11 The expression levels of enzyme-related genes.

10.7717/peerj.13427/supp-12 Supplemental Information 12 The expression levels of heat shock transcription factor.

10.7717/peerj.13427/supp-13 Supplemental Information 13 The expression levels of heat shock protein genes.

10.7717/peerj.13427/supp-14 Supplemental Information 14 Specific primers for differential gene sequences for qRT-PCR.

10.7717/peerj.13427/supp-15 Supplemental Information 15 Raw data of Water content.

10.7717/peerj.13427/supp-16 Supplemental Information 16 Raw data of physiological indexes.

## Additional Information and Declarations

### Data Availability

The following information was supplied regarding data availability: The raw RNA-Seq data are available at NCBI SRA: PRJNA663233.
