# Supplementary figures and images for: Heat stress response in Chinese cabbage (Brassica rapa L.) revealed by transcriptome and physiological analysis
Source: PeerJ. 2022 May 25;10:e13427. doi: 10.7717/peerj.13427 (PMC9147330; doi:10.7717/peerj.13427)

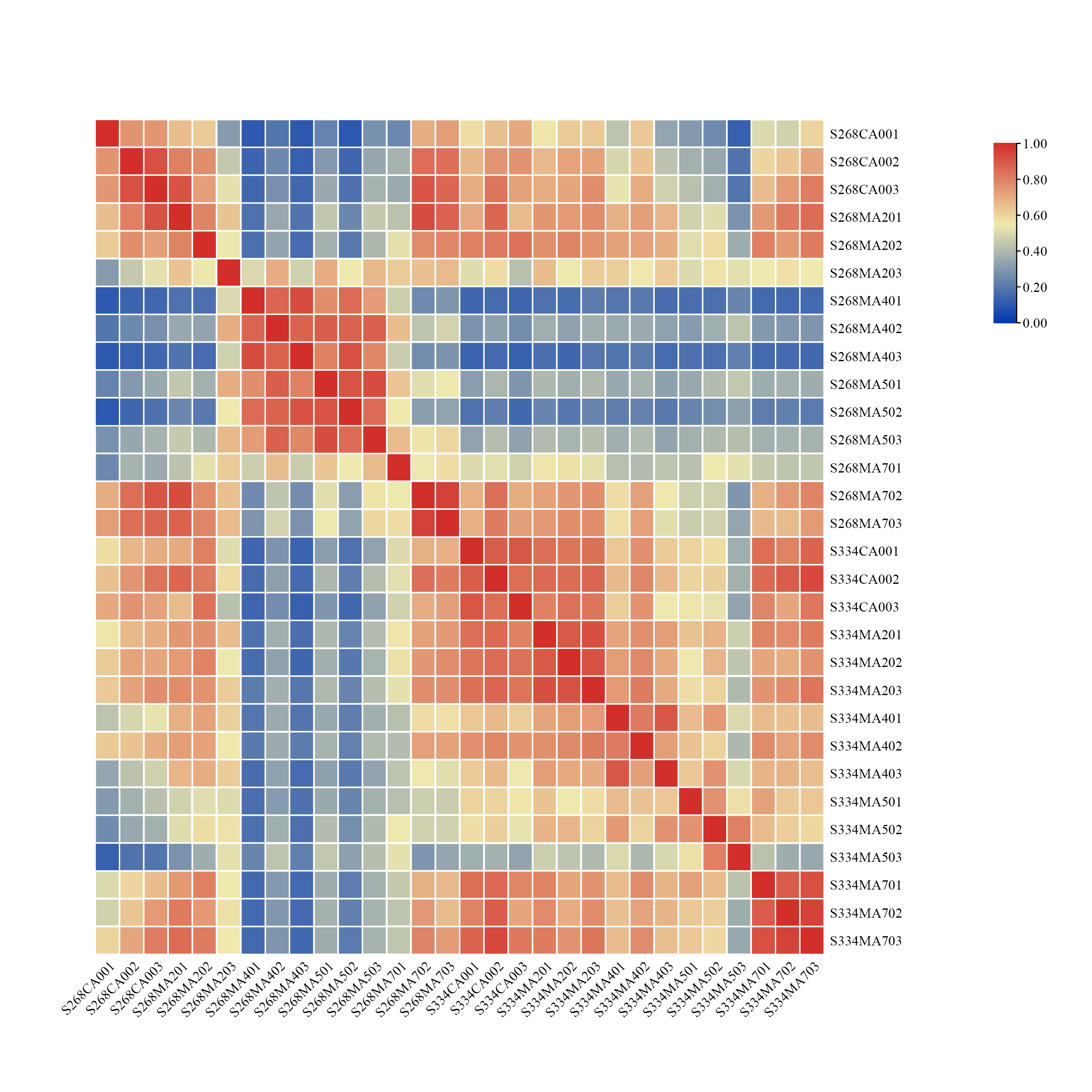

Supplement: Supplemental Information 1 — The color scale (red to blue) indicates correlation between samples [file peerj-10-13427-s001.png]
